# The authentication of Yanchi tan lamb based on lipidomic combined with particle swarm optimization-back propagation neural network

**DOI:** 10.1016/j.fochx.2024.102031

**Published:** 2024-11-22

**Authors:** Qi Yang, Dequan Zhang, Chongxin Liu, Le Xu, Shaobo Li, Xiaochun Zheng, Li Chen

**Affiliations:** Institute of Food Science and Technology, Chinese Academy of Agriculture Sciences, Key Laboratory of Agro-Products Quality and Safety Control in Storage and Transport Process, Ministry of Agriculture and Rural Affairs, Beijing 100193, China

**Keywords:** Tan lamb, Lipidomic, Food authenticity, Geographical indication, Chemometrics, Machine learning

## Abstract

This study successfully combined widely targeted lipidomic with a back propagation (BP) neural network optimized based on a particle swarm algorithm to identify the authenticity of Yanchi Tan lamb. An electronic nose and gas chromatography–olfactometry-mass spectrometry (GC-O-MS) were used to explore the flavor differences in Tan lamb from various regions. Among the 17 identified volatile compounds, 16 showed significant regional differences (*p* < 0.05). Lipidomic identified 1080 molecules across 41 lipid classes, with 11 lipids, including Carnitine 15:0, Carnitine 17:1, and Carnitine C8:1-OH, serving as potential markers for Yanchi Tan lamb. In addition, a stepwise linear discriminant model and three types of BP neural networks were used to identify the origin of Tan lamb. The results showed that particle swarm optimization-back propagation (PSO-BP) neural network had the best prediction effect, with 100 % prediction accuracy in both the training and test sets. The established PSO-BP model was able to achieve effective discrimination between Yanchi and non-Yanchi Tan lamb. These results provide a comprehensive perspective on the discrimination of Yanchi Tan lambs and improve the understanding of Tan lamb flavor and lipid composition in relation to origin.

## Highlights

• Identification of 1080 lipid compounds in two parts of Tan lamb from three regions.
• Eleven lipids can serve as taxonomic markers for Yanchi and non-Yanchi Tan lambs.
• The established PSO-BP model can achieve accurate discrimination of Yanchi Tan lamb.

## 1 Introduction

In recent years, there has been a significant focus on the geographic origin of food by government agencies, industry, scientists, and consumers worldwide (Bannor et al., 2023). High-quality products with geographical indications and appellations of origin are often associated with higher retail prices and greater market recognition compared to similar products (Zhao et al., 2022). Yanchi Tan lamb is one of China's geographical indications (GI) products. It is known for its tender and delicious meat, unique flavor, and is a result of the area's unique water, geographic environment, and climatic conditions (Liu et al., 2021). Yanchi Tan lamb possesses unique meat qualities that have led to its introduction in many other regions. It is now widely distributed in Ningxia Tongxin, Lingwu and other places, as well as in Gansu, Inner Mongolia and other areas adjacent to Ningxia. However, due to differences in ecological conditions and origin, these unique characteristics are not always maintained. The phenomenon of fraud and misuse of the Yanchi Tan lamb brand has greatly undermined its credibility and the rights and interests of consumers. Accurately distinguishing between different Tan lamb production areas is key to solving this problem.

As the “golden standard” of origin tracing, stable isotopes and mineral elements are widely used to identify the geographical origins of food products. In comparison to the aforementioned techniques, mass spectrometry-based omics technologies have rapidly advanced due to their advantages in high throughput, sensitivity, and selectivity (Hu et al., 2018). Omics technologies enable extensive exploration of food information, helping to more accurately identify distinguishing features of origin and improving the accuracy of traceability (Wood et al., 2008). Therefore, mass spectrometry-based omics technologies are employed in discerning the origins of food products such as wine (Phan & Tomasino, 2021), wheat (Jin et al., 2021), coffee (Aurum et al., 2022), and Coix seeds (Chen et al., 2023). These results demonstrate the significant potential of omics technologies in geographical origin identification. Lipids, as significant organic macromolecules, play a vital role in the food industry (Guo et al., 2023). As a key component of meat products, their composition and structure are influenced by environmental and climatic conditions (Chen et al., 2022). Wang et al. (2021) found differences in lipid profiles of beef from six countries including Argentina and Australia. Additionally, significant variations in the fatty acid composition of lambs raised in different grazing regions have been confirmed (Vasilev et al., 2020). Changes in lipid composition can directly reflect metabolic differences among different samples. Therefore, lipidomic focusing on lipids is utilized to identify the geographical origins of meat products, including tuna (Song, Chen, et al., 2020), beef (Robson et al., 2022), pork (Mi et al., 2019), and chicken (Tang et al., 2023). The strong performance of lipidomic technology in the field of food traceability can be attributed to the analysis of lipid molecular types and quantities in meat, providing data support for tracing origins. (Mahrous et al., 2023). Widely targeted lipidomic, which combines the high throughput of untargeted methods with the stability and accuracy of targeted methods (Chen, Fu, et al., 2024), helping to discover lipid biomarkers in Tan lamb from different regions and elucidating the link between biomarkers and origin. The lipid composition is closely linked to meat flavor (Wang et al., 2024; Xu et al., 2023). Investigating the lipidomics of Tan lamb not only aids in tracing its geographical origin but also lays the foundation for exploring flavor differences across regions.

Chemometrics provides multivariate statistical techniques developed for analytical chemistry, which have been widely adopted in the food industry. It provides important assistance in processing large and complex datasets generated by advanced analytical methods (Granato, Putnik, et al., 2018). The combination of omics and chemometrics in the traceability of the origin achieved good results (Hong et al., 2023), which provided ideas for the in-depth exploration of the connection between the lipidomic data and the origin of Tan lambs. The construction of the discriminant model is particularly important as the final step of this study. Both linear discriminant analysis methods (Esteki et al., 2018) and machine learning based methods (Song, Jiang, et al., 2020) have achieved better results in food certification. Among them, the stepwise linear regression discriminant is a model that only retains predictor variables with a significant effect on the target variable by gradually adding or removing variables. Due to its advantages of high interpretability and low overfitting risk, it has achieved good results in food origin traceability such as mutton (Liu et al., 2024), kiwi (Guo et al., 2017) and cuttlefish (Varrà et al., 2021). And the Back propagation (BP) neural network shows outstanding results in meat freshness prediction (Liu et al., 2023), Pericarpium Citri Reticulatae origin traceability (Chen, Xu, et al., 2024), and optimisation of extraction and purification process of flavonoid components in Astragalus (Xu et al., 2022), etc., provide the possibility of realising the accurate discrimination of Yanchi Tan lamb.

**Fig. 1:**
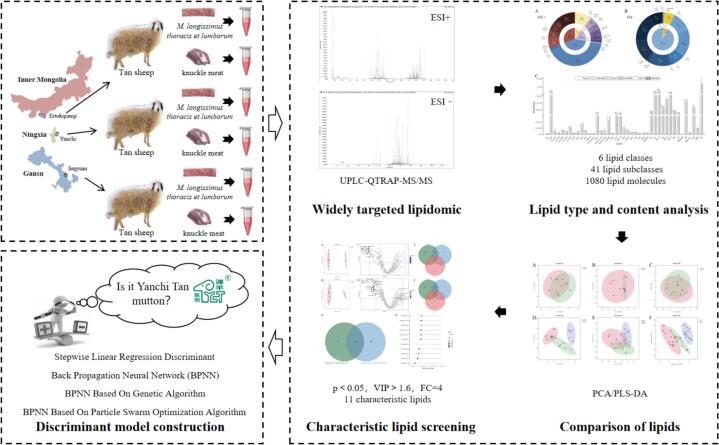
Process of Yanchi Tan lamb discrimination model construction based on widely targeted lipidomic combined with chemometrics.

This study investigated the differences in volatile compounds of Tan lamb from different regions using an electronic nose and headspace solid-phase microextraction-gas chromatography-olfactometry-mass spectrometry (HS-SPME-GC-O-MS). Additionally, it employed comprehensive targeted lipidomic to obtain lipidomic data of Tan lamb from various regions, using chemometrics to identify characteristic lipids of Yanchi Tan lamb. The feasibility of widely targeted lipidomic technology for discriminating Yanchi Tan lamb was assessed (The process of discriminative model construction is shown in Fig. 1). It provides a new idea for the origin certification of specific products with regional attributes, which is of great significance for protecting brand rights and interests and safeguarding consumer interests.

## 2 Materials and methods

### 2.1 Reagents

Lipid standards were purchased from Sigma-Aldrich or Avanti Polar Lipids (Alabaster, AL, USA). Chromatographic grade acetonitrile (ACN), methanol (MeOH), isopropanol (IPA), and methyl tertiary butyl methyl ether (MTBE) were purchased from Merck (Darmstadt, Germany). Formic acid (FA) was obtained from Sigma-Aldrich, while dichloromethane (CH_2_Cl_2_) and ammonium formate (AmFA) were supplied by Thermo Scientific. The experimental tests utilized ultrapure water produced by the Milli-Q system (Millipore, Bedford, MA, USA).

### 2.2 Sample collection

In 2023, samples of lamb were collected from Ningxia Hui Autonomous Region, Gansu Province and Inner Mongolia Autonomous Region, China. 6 individuals were selected from each region and the *M. longissimus thoracis et lumborum* and knuckle meat were collected. Details of the sampling information is shown in Table S1.

Muscle samples (1 g) were taken from sheep carcasses (6-month old, male) of the same batch of rearing and slaughtered sheep stored at 4 °C for 24 h in a constant temperature cold storage. The samples were prepared by removing the fascia and fat with a scalpel, then weighed and placed in a freezing tube. After being immersed in liquid nitrogen for 30 min, the samples were sent back to the laboratory on dry ice and stored at −80 °C until analysed.

### 2.3 Electronic nose analysis

2.0 g of sample was placed in a 20 mL headspace vial, equilibrated at 25 °C for 30 min and measured using a PEN3 (Airsense Analytics, Schwerin, Germany) portable Electronic nose analyser.

### 2.4 Volatile analysis

4.0 g of sample was mixed with 10 μL of internal standard solution (0.27 μg/μL 2-methyl-3-heptanone) in a 20 mL headspace vial and extracted using a DVB/CAR/PDMS extraction head. A Thermo Scientific ISQ LT Single Quadrupole Gas-Mass System with a DB-5 ms column (60 m × 0.25 mm i.d., 0.25 μm film thickness) was used for the analysis, with a helium flow rate of 0.8 mL/min. Headspace solid-phase micro extraction was resolved at 250 °C for 5 min, the initial heating temperature was maintained at 40 °C for 5 min, then increased to 200 °C at 5 °C/min, and finally increased to 250 °C at 15 °C/min and maintained at 250 °C for 8 min. The mass spectrometer was operated in EI mode at 70 eV, with an ion source temperature of 230 °C, a quadrupole temperature of 150 °C, a scanning range of 45.00–550.00 amu, and a solvent delay of 2.00 min. The compounds were separated by the column into the mass spectrometry detector with an olfactometer shunt ratio of 5:1.

### 2.5 Lipid extraction

The MTBE extraction method proposed by Matyash et al. (2008) was used with slight modifications. Accurately weigh 20 mg (±1 mg) of each sample and add 1 mL of lipid extract containing the internal standard (methyl tert-butyl ether: methanol = 3:1, *v*/v). Vortex (VORTEX-5, Kyllin-Bell, Haimen, Jiangsu Province, China) the mixture for 15 min, then add 200 μL of water and vortex for 1 min. Centrifuge the mixture for 10 min at 13500 g under the condition of 4 °C. The supernatant was evaporated with a vacuum concentrator after pipetting 200 μL. The extract was dissolved in 200 μL of a lipid complex solution (acetonitrile: isopropanol = 1:1, *v*/v). The solution was vortexed for 3 min and then centrifuged at 13500*g* for 3 min for LC-MS/MS analysis (ExionLC AD UPLC–QTRAP, SCIEX, Framingham, MA, USA).

### 2.6 Acquisition conditions for chromatography and mass spectrometry

The lipid classes and molecular species of Tan lamb were analysed using an ultra-high performance liquid chromatography system coupled to tandem mass spectrometry. The sample was separated using a Thermo Accucore™ C30 column with an inner diameter of 2.1 mm and a length of 100 mm, packed with 2.6 μm particles. The mobile phase consisted of two solvents: A and B. Solvent A was composed of a mixture of acetonitrile and water in a ratio of 60:40 (*v*/v) with 0.1 % formic acid and 10 mmol/L ammonium formate. Solvent B was composed of a mixture of acetonitrile and isopropanol in a ratio of 10:90 (v/v) with 0.1 % formic acid and 10 mmol/L ammonium formate. Gradient program, the A/B (80:20, v/v) at 0 min, A/B (70:30, v/v) at 2 min, A/B (40:60, v/v) at 4 min, A/B (15:85, v/v) at 9 min, A/B (10:90, v/v) at 14 min, A/B (5:95, v/v) at 15.5 min, A/B (5:95, v/v) at 17.3 min, A/B (80:20, v/v) at 17.5 min, A/B (80:20, v/v) at 20 min; the flow rate was set to 0.35 mL/min, the column temperature was maintained at 45 °C, and the injection volume was 2 μL. The effluent was connected to an electrospray ion source triple quadrupole linear ion trap mass spectrometer (ESI-QTRAP-MS). The electrospray ion source temperature was set to 500 °C. The mass spectrometry voltage was set to 5500 V in positive ion mode and − 4500 V in negative ion mode. The ion source gas1 was set to 45 psi, gas2 to 55 psi, and air curtain gas to 35 psi. The collision-induced ionization parameter was set to Medium. In the triple quadrupole, each ion pair was scanned and detected based on optimized de-clustering voltage and collision energy.

### 2.7 Data processing

The raw peak intensity data (*.wiff) was extracted from Analyst® software (SCIEX, Framingham, MA, USA) using MultiQuant 3.0.3 software (SCIEX, Framingham, MA, USA). The peaks in the MRM chromatograms were extracted using the following parameters: Gaussian Smooth Width (0 points), RT Half Windows (30 s), Min.Peak Width (2 points), and Min.Peak Height (0). Baseline Noise Percentages were set to 70 % and Baseline Subtraction was applied. The chromatographic peak filtering had a minimum intensity of 1000 cps, a minimum signal-to-noise ratio of 5, and a retention time deviation of less than 0.2 min. Substances with a detection rate of less than 50 % and a coefficient of variation of more than 0.3 were removed based on the peak area of quality control (QC) samples, resulting in a final list of substances.

### 2.8 Data analysis

Data analysis was performed using MetaboAnalyst 5.0 (https://www.metaboanalyst.ca). The quality of the samples was initially evaluated using principal components analysis (PCA) (Fig. S1). Subsequently, differences between various sources of Tan sheep were determined by combining PCA with partial least squares discriminant analysis (PLS-DA). The study used independent samples *t*-test and fold change (FC) analysis, combined with variable importance in projection (VIP) obtained by orthogonal partial least squares discriminant analysis (OPLS-DA), to identify potential lipid markers in different regions of Tan lamb. The screening conditions were set at *p* < 0.05, a critical value of FC = 4 (FC > 4 or < 0.25), and VIP > 1.6. Screening was performed using R software (version 3.5.1). The results were displayed using Venn and lollipop plots. The biomarker data was imported into the SPSS 26.0 software package and screened using stepwise regression linear discriminant analysis. A linear discriminant model was created to accurately determine whether the lamb is Yanchi Tan or not.

### 2.9 Construction of machine learning classification models

Three types of BP neural networks (BP, genetic algorithm (GA)-BP, particle swarm optimization (PSO)-BP) were used to discriminate whether the lamb samples were from Yanchi or not, based on the potential lipid markers obtained from screening of Tan lamb from different regions. The analysis process for all methods was conducted using MATLAB R2023b. The model parameters are set as follows:

BP: The data is randomly divided into training and test sets in a 4:1 ratio. The input layer comprises 11 nodes, the hidden layer 6 nodes, and the output layer 2 nodes.

GA: The number of genetic generations is 30, the population size is 5, the number of optimisation parameters is S, where S is the sum of the number of input nodes, the number of hidden layer nodes, the number of output layer nodes, and the number of hidden layer nodes, and the optimisation variable boundaries are from −2.0 to 2.0.

PSO: The number of population updates was 30, the population size was 5, the maximum velocity was 1.0, the minimum velocity was −1.0, and the optimisation variables were constrained between −2.0 and 2.0.

The precision, recall, accuracy, error rate and F1 score of the model were calculated using the true positives (TP), false negatives (FN), false positives (FP), and true negatives (TN) from the confusion matrix to evaluate the model's performance. Each metric was computed using the following formulas:

(1) $$\mathrm{Precision}=\frac{\mathrm{TP}}{\mathrm{TP}+\mathrm{FP}}$$

(2) $$\mathrm{Recall}=\frac{\mathrm{TP}}{\mathrm{TP}+\mathrm{FN}}$$

(3) $$\mathrm{Accuracy}=\frac{\mathrm{TP}+\mathrm{TN}}{\mathrm{TP}+\mathrm{FP}+\mathrm{TN}+\mathrm{FN}}$$

(4) $$\mathrm{Error rate}=\frac{\mathrm{FP}+\mathrm{FN}}{\mathrm{TP}+\mathrm{FP}+\mathrm{TN}+\mathrm{FN}}$$

(5) $$F1\;\mathrm{Score}=\frac{2\mathrm{TP}}{2\mathrm{TP}+\mathrm{FP}+\mathrm{FN}}$$

## 3 Results and discussion

### 3.1 Analysis of volatile flavouring compounds in tan lamb from different regions

**Fig. 2:**
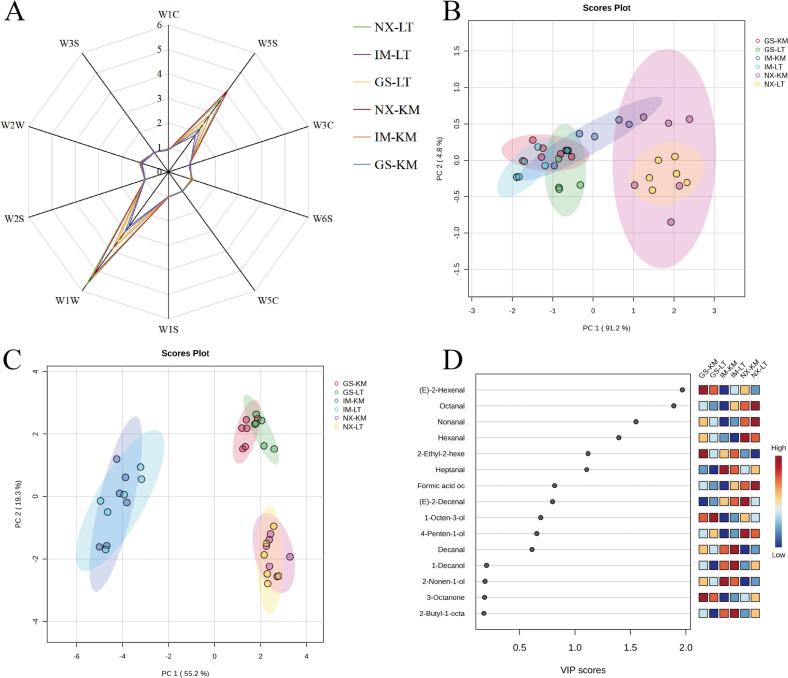
Electronic nose radar plot (A) and principal component analysis (B) for Tan lamb from different regions; principal component analysis (C) and VIP score (D) for volatile flavor compounds in Tan lamb from different regions (NX-LT, *M. longissimus thoracis et lumborum* of Yanchi Tan lamb, Ningxia; NX-KM, knuckle meat of Yanchi Tan lamb, Ningxia; GS-LT, *M. longissimus thoracis et lumborum* of Jingyuan Tan lamb, Gansu; GS-KM, knuckle meat of Jingyuan Tan lamb, Gansu; IM-LT, *M. longissimus thoracis et lumborum* of Ertokqianqi Tan lamb, Inner Mongolia; IM-KM, knuckle meat of Ertokqianqi Tan lamb, Inner Mongolia; *n* = 6).

Electronic noses are commonly used to resolve the odor profiles of food products, and as shown in Fig. 2A, the radargram profiles of the lamb samples were similar, with the W1W (terpenes and sulfur-containing organic compound) and W5S (broad-range nitrous oxides) sensors strongly corresponded to the Tan lamb samples, but the intensity of the sensors varied among the samples. The similarity of the odor profiles of the Tan lamb samples was further observed using principal component analysis (PCA), which showed that the projections of the samples from different parts of the same region overlapped severely, and the Tan lamb from the same region had similar odors. It is noteworthy that the projections of Gansu and Inner Mongolia samples overlapped each other, while the Yanchi Tan lamb samples from Ningxia were separated from the other two regions, suggesting that in terms of odor profiles, Yanchi Tan lamb differed significantly from the other two regions (Fig. 2B). To further resolve the differences in volatile flavor substances of Tan lamb from different regions, HS-SPME-GC-O-MS was used. A total of 17 volatile compounds were identified in all samples (Table S2), and a total of 16 volatile compounds differed significantly (*p* < 0.05) in different regions. The PCA downscaling revealed that the Tan lamb samples showed regional aggregation, and the aggregation of samples from the same region was obvious (Fig. 2C). Compared with the electronic nose, the volatile compounds were more effective in distinguishing Tan lamb from the three regions, and the specific flavor compounds were more representative of the volatile flavor profile of Tan lamb than the generalized odor profile. The key volatile compounds for distinguishing the origin of Tan lamb were screened by partial least squares discriminant analysis (PLS-DA), and (E)-2-hexenal (green), octanal (fatty), and nonanal (fatty) (VIP>1.5) played important roles in distinguishing the Tan lambs from different regions (Fig. 2D). In summary, different origins lead to differences in the volatiles of Tan lamb. Therefore, it is necessary to discriminate the origin of Tan lamb.

### 3.2 Analysis of the lipid content and category composition of tan lamb from various regions

**Fig. 3:**
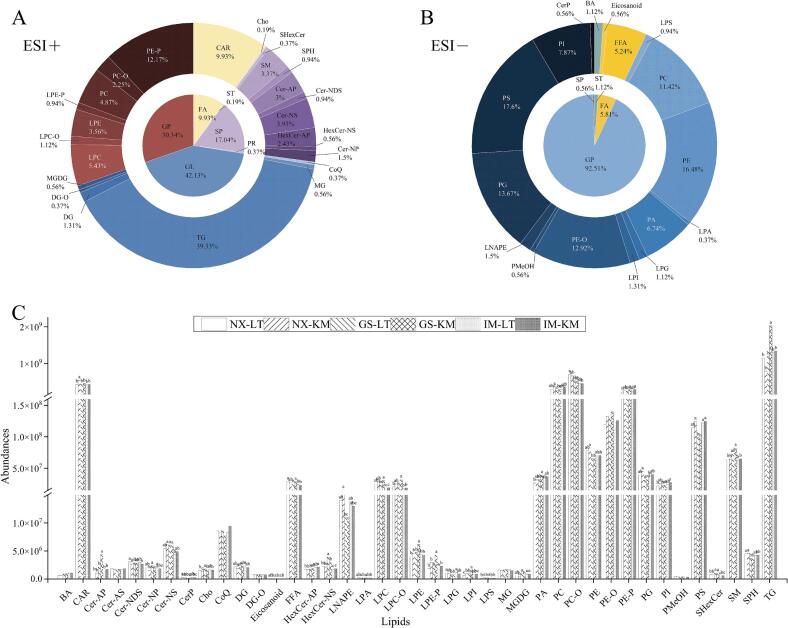
Asahi diagrams of lipid species identified in ESI+ (A) and ESI- (B) mode; (C) Abundance of different origins and parts of Tan lamb on each lipid class (NX-LT, *M. longissimus thoracis et lumborum* of Yanchi Tan lamb, Ningxia; NX-KM, knuckle meat of Yanchi Tan lamb, Ningxia; GS-LT, *M. longissimus thoracis et lumborum* of Jingyuan Tan lamb, Gansu; GS-KM, knuckle meat of Jingyuan Tan lamb, Gansu; IM-LT, *M. longissimus thoracis et lumborum* of Ertokqianqi Tan lamb, Inner Mongolia; IM-KM, knuckle meat of Ertokqianqi Tan lamb, Inner Mongolia; n = 6).

This work presents a widely targeted lipidomic analysis of Tan lamb from different regions using UPLC-MS/MS in positive and negative ion models (Fig. S2). Overall, a total of 1080 lipids were detected, belonging to 41 subclasses across six categories: glycerolipids (GL), glycerophospholipids (GP), sphingolipids (SP), fatty acids (FA), prenol lipids (PR) and sterol lipids (ST). In the positive ion mode, a total of 546 lipid species belonging to 24 lipid subclasses were detected in 6 lipid categories mentioned above. Among them, the content of GL and GP accounted for 41.21 % and 29.67 % of the total lipids, respectively. Herein, the content of Triglycerides (TG) accounted for 39.33 % of the total lipids which was the highest percentage of lipids in all of identified lipid subclasses (Fig. 3A). In the negative ion mode, a total of 534 lipids were detected, categorized into 18 subclasses under the four categories: GP, FA, ST, and SP. Especially, GP accounted for the largest proportion in lamb, representing 92.51 % of the total lipids in the negative ion mode (Fig. 3B). Then, the content of lipid subclasses was performed (Fig. 3C), and result revealed that TG, O-phosphatidylcholine (PC-O), Carnitine (CAR), PC, and P- phosphatidylethanolamine (PE-P) were the most abundant lipid subclasses in Tan lamb, in line with results of Li et al. (2022) that reported TG and PC lipids were the most prevalent lipids in terms of type and content of lipids. TG is one of the primary forms of fat, and its abundance in lamb is the primary reason for the formation of higher levels of TG. PC is a phospholipid-like substance that is mainly found in cell membranes and is involved in biological processes such as cell membrane composition and signalling. It is widely present in animal tissues (Pan et al., 2023). Compared to Jia et al.'s (2021) detection of lipids in Tan lamb, the use of a lipid extraction method with widely targeted lipidomic techniques in this study may have led to the detection of more lipid species.

A total of 34 lipids from different regions of Tan lamb showed significant differences (*p* < 0.05) (Fig. 3C). Significant differences were observed in CAR, dihydrosphingosine (Cer-NDS), Cholesterol (Cho), lysophosphatidylinositol (LPI), and lysophosphatidylserine (LPS) among different parts of Yanchi Tan lamb from the same region. Similarly, phytosphingosine (Cer-AP), phytosphingosine (Cer-NP), Cho, Eicosanoid, free fatty acid (FFA), non-hydroxyfatty acid-sphingosine (HexCer-NS), lysophosphatidic acid (LPA), lysophosphatidylcholine (LPC), O-lysophosphatidylcholine (LPC-O), and lysophosphatidylethanolamine (LPE) showed significant differences among different parts of Gansu Tan lamb. In Inner Mongolian Tan lamb, significant differences were observed in Eicosanoid, P-lysophosphatidylethanolamine (LPE-P), LPI, LPS, monogalactosyldiacylglycerol (MGDG) and TG among different parts. The levels of three types of lipids, Cho, LPI, and LPS, varied significantly among different parts of Yanchi Tan lamb and Gansu Tan lamb. Differential lipids between Gansu Tan lamb and Inner Mongolia Tan lamb sites all included Eicosanoid, while no lipid shows significant differences across different sites in all three regions. In terms of the same parts from different regions, only the Tan lamb *M. longissimus thoracis et lumborum* from all three regions were significantly different in sulfatides (SHexCer), whereas no relevant lipid subclasses were significantly different in all three regions of knuckle muscles (Table S3).

Structural and functional differences in muscle tissues are one of the key factors contributing to differences in lipid composition in different parts of the animal (Li et al., 2021). Specifically, those muscle tissues that are primarily responsible for supportive and athletic functions usually have fewer fat deposits. This is because these muscle areas exhibit higher metabolic rates due to frequent activity, which reduces fat accumulation and storage. This phenomenon reflects the influence of muscle fibre type and activity level on lipid metabolism. Additionally, the impact of rearing methods and nutritional conditions on fat distribution in Tan sheep muscle cannot be overlooked. The use of high-energy feeds may cause an increase in fat in certain muscle areas, particularly in those that are less active. This trend of fat accumulation is influenced not only by the energy level of the feed but also by the proportion of specific nutrients in the feed.

Consistent with the work of Vasilev et al. (2019), environment is one of the key factors influencing the lipid composition of meat. The environment in which animals grow and the natural feed resources available to them are directly affected by climatic characteristics, geography, and seasonal variations in various regions. These factors also influence the content and specific composition of lipids. In addition, different feeding management and nutritional conditions can also cause lipid differences by affecting energy and fat metabolism (Zhao et al., 2023). In the Ningxia region, grazing activities are restricted due to arid climatic conditions and corresponding environmental policies. In contrast, in the other two regions, Tan lambs are reared through a combination of natural grazing and housed feeding. This difference in feeding patterns may be the primary reason for the variations in lipid composition of Tan lambs across different regions.

### 3.3 Comparison of tan lamb lipids from various regions

**Fig. 4:**
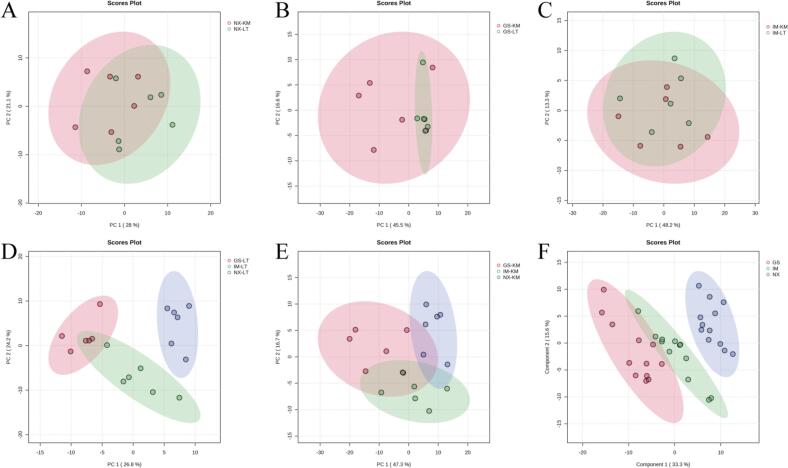
Principal components analysis (PCA) score plots of different parts (A, B, C) and different regions (D, E) of Tan lamb(n = 6); Plot of partial least squares discriminant analysis (PLS-DA) (F); scores of Tan lamb from different regions (NX, Yanchi Tan lamb, Ningxia; GS, Jingyuan Tan lamb; IM, Ertokqianqi Tan lamb, Inner Mongolia; *n* = 12).

To further compare the lipidomic characteristics of Tan lamb from different regions, multivariate statistical analysis was used to explore the differences in lipids of Tan lamb samples. The PCA method is an unsupervised pattern recognition technique that offers an initial understanding of the lipid differences between groups of samples and the variability between samples within groups (Granato, Santos, et al., 2018). Figs. 4A-C demonstrate the differences between Tan lamb samples from three regions: Yanchi Tan lamb from Ningxia, Jingyuan Tan lamb from Gansu, and Ertokqianqi Tan lamb from Inner Mongolia, in relation to the two parts of *M. longissimus thoracis et lumborum* and knuckle muscles. The PCA scoring plots revealed only a partial separation between the *M. longissimus thoracis et lumborum* and knuckle muscles of the Tan lamb samples from three different regions. This suggests a high degree of lipid similarity among the samples from different parts of the same region. This presents consistency with the results obtained by Liu et al. (2024) on the study of lipids from different parts of Bahan crossbreed sheep and Tan lamb. Fig. 4D and E show the differences between samples of Tan lamb from the same part in different regions. Fig. 4E displays the grouping of knuckle meat by principal components 1 (PC1) and 2 (PC2) in three regions: Yanchi, Ningxia; Jingyuan, Gansu; and Ertokqianqi, Inner Mongolia. The projections of knuckle meat samples from the three regions on principal components 1 and 2 only partially overlap. PC1 and PC2 explain 47.3 % and 16.7 % of the total variation, respectively. It is worth noting that the *M. longissimus thoracis et lumborum* samples of Yanchi Tan lamb from Ningxia could be completely distinguished from those of the other two regions, while some overlap still existed between the *M. longissimus thoracis et lumborum* samples from Gansu and Inner Mongolia. This indicates that there are some differences in lipids between the samples of Yanchi Tan lamb from different regions, and the differences in lipids between Yanchi Tan lamb and the samples of the other two regions are more obvious. To further investigate the differences in lipid quality of Tan lamb from different regions, supervised PLS-DA was performed on Tan lamb samples from three regions (Lee et al., 2018). The PLS-DA scoring plot showed that Gansu and Inner Mongolia Tan lamb samples still partially overlapped, while good separation was obtained between Yanchi Tan lamb and non-Yanchi Tan lamb samples (Fig. 4F), which further promotes the subsequent screening and discrimination of Yanchi Tan lamb characteristic lipids.

### 3.4 Lipid screening for Yanchi tan lamb characterization

**Fig. 5:**
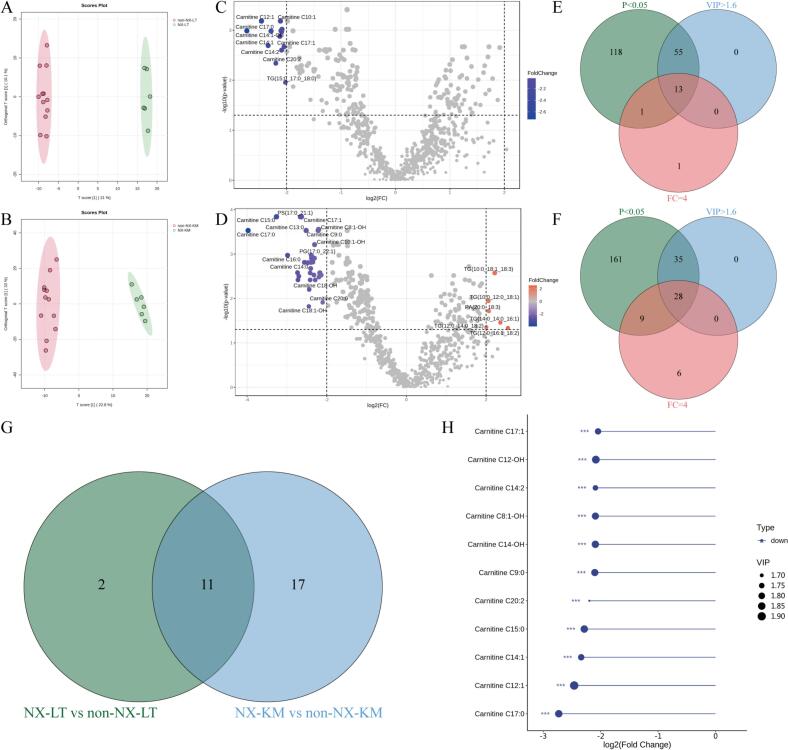
Orthogonal partial least squares discriminant analysis (OPLS-DA) score plots (AB), volcano plots (CD) and Venn plots (EFG) were used to screen potential markers for Yanchi Tan lamb and to identify them as Yanchi Tan lamb, and lollipop plots (H) were used to demonstrate the screened markers (non-NX-LT, *M. longissimus thoracis et lumborum* of non-Ningxia Yanchi Tan lamb; non-NX-KM, knuckle meat of non- Ningxia Yanchi Tan lamb; n = 12).

To identify potential lipid biomarkers for effectively differentiating Yanchi Tan lamb from non-Yanchi Tan lamb, we first performed differential lipid molecular screening on the *M. longissimus thoracis et lumborum* of the Tan lamb. The OPLS-DA model effectively differentiated between Yanchi Tan lamb and non-Yanchi Tan lamb, as shown in Fig. 5A. Further screening was conducted on the lipid molecules with VIP values greater than 1.6, which were selected as differential lipids. Volcano plots were used to display the differential lipids between Yanchi Tan lambs and non-Yanchi Tan lambs, based on the results obtained from an independent samples *t*-test. Fourteen lipids were identified as down-regulated by applying the criteria of *p* < 0.05 and FC critical value = 4 (Fig. 5C). The results of the differential lipid screening of the *M. longissimus thoracis et lumborum* of Tan lamb were presented in a Venn diagram, yielding 13 lipid compounds to be used as further studies (Fig. 5E).

To determine the representativeness of the screened differential lipids, Tan lamb knuckle meat was selected for further validation. The OPLS-DA model effectively differentiated Yanchi Tan lambs from non-Yanchi Tan lambs (Fig. 5B). Lipid molecules with VIP values greater than 1.6 were further screened, resulting in the selection of 6 up-regulated lipids and 31 down-regulated lipids with a *p* < 0.05 and FC threshold = 4 (Fig. 5D). A Venn diagram was used to obtain 28 lipid compounds (Fig. 5F). The differential lipid compounds obtained from the *M. longissimus thoracis et lumborum* and knuckle meat of Tan lamb were analysed. Eleven differential lipid compounds were identified as characteristic lipids that can distinguish Yanchi from non-Yanchi Tan lamb (Fig. 5G). The 11 identified lipids were illustrated in a lollipop plot (Fig. 5H). All of these lipids were classified as Carnitine, including C17:1, C12-OH, C14:2, C8:1-OH, C14-OH, C9:0, C20:2, C15:0, C14:1, C12:1, and C17:0. Notably, all 11 characteristic lipids are downregulated with highly significant differences (*p* < 0.001). The content of the 11 carnitines in Yanchi Tan lamb was significantly higher than in non-Yanchi Tan lamb. Finally, the 11 potential lipid biomarkers identified were utilized to develop a discriminant model for Yanchi Tan lamb (Table 1). Information on formulae, molecular weights and ionization models are also presented in the table.

**Table 1:** Identification of potential lipid markers in Yanchi Tan lamb lambs.

Lipid Code	Compounds	Formula	Theoretical *m*/*z*	Observed m/z	Ionization model	Lipid Category	NX-LT	NX-KM	non-NX-LT	non-NX-KM
**CAR1**	**Carnitine C15:0**	**C22H43NO4**	**385.3192**	**386.3250**	**[M** **+** **H]+**	**Fatty Acyls**	**7.51** **×** **10**^**5**^ **±** **1.21** **×** **10**^**5**^**b**	**1.07** **×** **10**^**6**^ **±** **2.12** **×** **10**^**5**^**a**	**1.45** **×** **10**^**5**^ **±** **3.45** **×** **10**^**4**^**c**	**1.27** **×** **10**^**5**^ **±** **1.43** **×** **10**^**4**^**c**
**CAR2**	**Carnitine C17:1**	**C24H45NO4**	**411.3348**	**412.3427**	**[M** **+** **H]+**	**Fatty Acyls**	**1.43** **×** **10**^**5**^ **±** **2.15** **×** **10**^**4**^**a**	**1.88** **×** **10**^**5**^ **±** **4.10** **×** **10**^**4**^**a**	**3.38** **×** **10**^**4**^ **±** **5.78** **×** **10**^**3**^**b**	**3.67** **×** **10**^**4**^ **±** **4.23** **×** **10**^**3**^**b**
**CAR3**	**Carnitine C8:1-OH**	**C15H27NO5**	**301.1889**	**302.1975**	**[M** **+** **H]+**	**Fatty Acyls**	**5.23** **×** **10**^**5**^ **±** **9.79** **×** **10**^**4**^**b**	**7.49** **×** **10**^**5**^ **±** **8.40** **×** **10**^**4**^**a**	**1.19** **×** **10**^**5**^ **±** **1.73** **×** **10**^**4**^**c**	**2.04** **×** **10**^**5**^ **±** **2.88** **×** **10**^**4**^**c**
CAR4	Carnitine C17:0	C24H47NO4	413.3505	414.3583	[M + H]+	Fatty Acyls	2.67 × 10^6^ ± 4.94 × 10^5^b	4.74 × 10^6^ ± 1.18 × 10^6^a	3.89 × 10^5^ ± 1.59 × 10^5^c	3.24 × 10^5^ ± 6.01 × 10^4^c
**CAR5**	**Carnitine C9:0**	**C16H31NO4**	**301.2253**	**302.2331**	**[M** **+** **H]+**	**Fatty Acyls**	**7.01** **×** **10**^**5**^ **±** **1.28** **×** **10**^**5**^**b**	**1.02** **×** **10**^**6**^ **±** **1.15** **×** **10**^**5**^**a**	**1.58** **×** **10**^**5**^ **±** **2.32** **×** **10**^**4**^**c**	**2.76** **×** **10**^**5**^ **±** **3.83** **×** **10**^**4**^**c**
CAR6	Carnitine C12:1	C19H35NO4	341.2566	342.2654	[M + H]+	Fatty Acyls	3.05 × 10^5^ ± 2.72 × 10^4^a	3.94 × 10^5^ ± 1.06 × 10^5^a	5.32 × 10^4^ ± 1.05 × 10^4^b	9.13 × 10^4^ ± 1.07 × 10^4^b
CAR7	Carnitine C20:2	C27H49NO4	451.3662	452.3735	[M + H]+	Fatty Acyls	1.53 × 10^5^ ± 2.84 × 10^4^b	2.81 × 10^5^ ± 6.67 × 10^4^a	3.28 × 10^4^ ± 6.76 × 10^3^c	5.25 × 10^4^ ± 9.98 × 10^3^c
CAR8	Carnitine C14:1	C21H39NO4	369.2879	370.2968	[M + H]+	Fatty Acyls	1.88 × 10^6^ ± 3.00 × 10^5^a	2.07 × 10^6^ ± 7.15 × 10^5^a	3.64 × 10^5^ ± 9.52 × 10^4^b	4.40 × 10^5^ ± 6.88 × 10^4^b
CAR9	Carnitine C14:2	C21H37NO4	367.2722	368.2811	[M + H]+	Fatty Acyls	1.10 × 10^5^ ± 2.07 × 10^4^a	1.20 × 10^5^ ± 3.45 × 10^4^a	2.53 × 10^4^ ± 5.08 × 10^3^b	3.24 × 10^4^ ± 5.356 × 10^3^b
CAR10	Carnitine C12-OH	C19H37NO5	359.2672	360.2760	[M + H]+	Fatty Acyls	2.47 × 10^5^ ± 1.15 × 10^4^b	4.83 × 10^5^ ± 1.46 × 10^5^a	5.59 × 10^4^ ± 1.03 × 10^4^d	1.24 × 10^5^ ± 1.97 × 10^4^bc
CAR11	Carnitine C14-OH	C21H41NO5	387.2985	388.3074	[M + H]+	Fatty Acyls	1.13 × 10^5^ ± 6.46 × 10^3^b	2.49 × 10^5^ ± 9.60 × 10^4^a	2.50 × 10^4^ ± 5.54 × 10^3^b	5.56 × 10^4^ ± 9.67 × 10^3^b

Note: Each value is expressed as Mean ± Standard Error (S.E.); a-d Different letters indicate statistically significant differences based on a Duncan test at a level of significance of *p* < 0.05; The lipids highlighted in bold were included in the established discriminant model.

The unique growth environment and rearing methods might be why characteristic carnitines are at higher levels in Yanchi Tan lamb. The high concentration of saline-alkali soil and water in the salt flats forces higher carnitine metabolism. Additionally, Yanchi Tan lamb might consume more saline-alkali plants during rearing, increasing carnitine intake. Carnitine plays a positive role in human health and function. It promotes the conversion and oxidation of fatty acids, aiding in the conversion of fat into energy (Del Vecchio et al., 2017). Additionally, it regulates blood pressure and blood glucose balance, reducing the risk of chronic diseases such as heart disease and diabetes (Zamani et al., 2023). Furthermore, it has been shown to improve athletic performance (Fielding et al., 2018). Yanchi Tan lamb is rich in pentadecenoyl carnitine, which has been found to promote endocannabinoids, support serotonin, and contain antihistamines. These activities are effective in regulating inflammation, improving mood, sleep, and reducing stress (Venn-Watson et al., 2022).

### 3.5 Stepwise linear discriminant model construction

Stepwise discriminant analysis was used to establish a discriminant model for Yanchi Tan lamb using 11 lipid biomarkers (CAR1-CAR11). Table S4 shows the stepwise screening process of the model. The new discriminant model is obtained by gradually removing the parameter with the lowest F-value. The model's variance was kept at 100 % across different numbers of factors. As the number of factors decreased during the stepwise discrimination process, the typical correlation of the model decreased from 0.945 to 0.826. Additionally, the model's prediction accuracy and cross-validation accuracy showed a trend of first decreasing, then increasing, and finally decreasing again. As the number of factors decreases, the model may lose some information, causing the model to decrease in relevance. At the same time, the model becomes simpler and also reduces the interference of overfitting and noise, which improves the predictive performance of the model, but the removal of too many factors may result in the loss of important information, which in turn results in a decrease in model performance. The model with fewer factors but higher discriminative accuracy was chosen for distinguishing Yanchi Tan lamb. Thus, factors CAR1, CAR2, CAR3, and CAR5 were ultimately retained. The discriminant function for the Yanchi Tan lamb samples, as proposed by Fisher, is presented below:

(6) $$Y_{\mathrm{NX}\;\mathrm{Tan}\;\text{Sheep}}=4.41\times 10^{-6}\;\text{CAR1}+2.98\times 10^{-5}\;\text{CAR2}+3.54\times 10^{-5}\;\text{CAR3}–1.14\times 10^{-5}\;\text{CAR5}–11.501$$

(7) $$Y_{\text{non}-\mathrm{NX}\;\mathrm{Tan}\;\text{Sheep}}=2.86\times 10^{-5}\;\text{CAR2}–3.93\times 10^{-6}\;\text{CAR1}+1.58\times 10^{-5}\;\text{CAR3}–7.33\times 10^{-6}\;\text{CAR5}–1.407$$

Where CAR1 is Carnitine C15:0, CAR2 is Carnitine C17:1, CAR3 is Carnitine C8:1-OH, and CAR5 is Carnitine C9:0. The lipid content of the lamb sample, which was previously unknown, is inserted into the discriminant function mentioned above. By comparing the Y values obtained from the two functions, the larger Y value is considered as the discriminant result.

The discriminant model achieved a predictive accuracy of 97.2 % and a cross-validation accuracy of 94.4 %. Overall, the stepwise linear discriminant model achieves good results in determining the origin of Yanchi Tan lamb.

### 3.6 BP neural network models construction

**Fig. 6:**
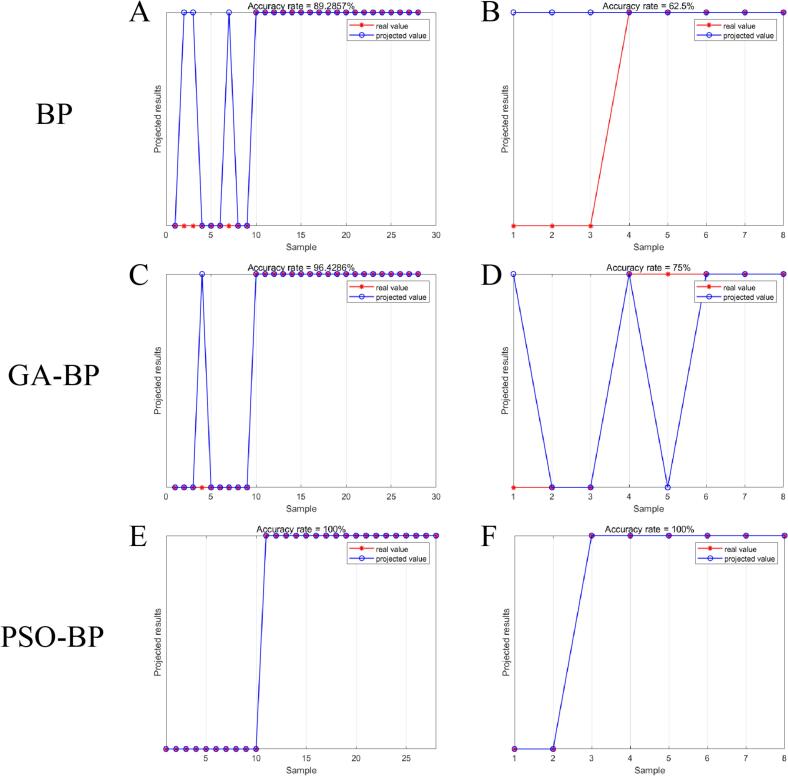
The accuracy rate of the three back propagation neural network training (A, C, E) and test (B, D, F) sets (The 36 samples were randomly divided into a training set (28) and a test set (8) in a 4:1 manner).

In addition, three types of BP neural networks were used to further explore the possibility of applying machine learning classification methods to distinguish Yanchi Tan lamb. The BP neural network demonstrated good performance in discriminating lamb breeds and parts in the study by Liu et al. (2024). However, on the discrimination between Yanchi and non-Yanchi Tan lambs in this study, the classification model constructed by BP neural network had a prediction accuracy of 89.3 % in the training set (Fig. 6A) and 62.5 % in the test set (Fig. 6B). The effect of differences in origin on lipids to a different extent than the effect of variety or site on lipids may be one of the factors contributing to the poor performance of the BP neural network in this study. The selection of parameters such as weights and biases in machine learning methods is crucial in determining the model's predictive performance. To improve the predictive performance of the BP neural network, we optimized its parameters using GA and PSO. By comparing the prediction accuracy of three types of BP neural networks, it can be observed that the optimized BP neural network shows varying degrees of improvement compared to the single BP neural network. The GA-BP neural network model achieves an increase in the prediction accuracy of the training set to 96.4 % (Fig. 6C) and an increase in the prediction accuracy of the test set to 75 % (Fig. 6D). The PSO-BP neural network model has an even greater improvement in prediction performance, with 100 % prediction accuracy in both the training and test sets (Fig. 6E, F). The model performance was further evaluated by precision, accuracy, recall, error rate and F1 value, and the optimized model showed improvements across all these metrics (Table S5). Overall, the PSO-BP model demonstrates the most outstanding performance.

Compared to the model training process of a single BP neural network, the PSO algorithm has the advantage of avoiding local optima and is more likely to find solutions closer to the global optimum (Ma et al., 2024). This advantage may be the key to significantly enhancing predictive performance after optimizing the BP neural network with the PSO algorithm. Furthermore, in comparison with the GA-BP model, the PSO-BP model demonstrates superior performance, possibly due to the differences in their optimization approaches. Unlike the GA, the PSO algorithm possesses a characteristic of memory that enables all particles to retain good solution knowledge. In addition, the GA optimisation process shares information between chromosomes, and the PSO algorithm's search process is more independent compared to the GA (Zhang et al., 2024). This distinction may also be a crucial factor contributing to the performance differences between the two algorithms.

## 4 Conclusions

In this study, we confirmed that geographical factors cause differences in the volatile flavor compounds of Tan lamb. (E)-2-hexenal, octanal, and nonanal (VIP > 1.5) play important roles in distinguishing Tan lamb from different regions. We further compared lipidomic differences between Yanchi and non-Yanchi Tan lamb, demonstrating the feasibility of using lipidomic combined with machine learning for identification. Multivariate statistical analysis allowed us to select 11 potential lipid markers from 1080 identified molecules to differentiate meat origin. When comparing the stepwise linear discriminant model with three machine learning methods, the PSO-BP method showed the best performance for Yanchi Tan lamb identification, achieving 100 % prediction accuracy for both training and test sets. This indicates that the PSO-BP model can accurately distinguish Yanchi Tan lamb. These insights will aid in enhancing quality control and market regulation of Tan lamb. Future research could use multi-omics approaches to further explore the impact of regional differences on meat quality, promoting the development of high-quality Tan lamb.

## CRediT authorship contribution statement

**Qi Yang:** Writing – original draft, Visualization, Methodology, Investigation, Formal analysis, Data curation. **Dequan Zhang:** Writing – review & editing, Supervision, Resources, Funding acquisition, Data curation. **Chongxin Liu:** Writing – review & editing, Methodology, Data curation. **Le Xu:** Writing – review & editing, Methodology, Data curation. **Shaobo Li:** Writing – review & editing, Software, Conceptualization. **Xiaochun Zheng:** Resources. **Li Chen:** Writing – review & editing, Project administration, Investigation, Formal analysis, Data curation.

## Declaration of competing interest

The authors declare that they have no known competing financial interests or personal relationships that could have appeared to influence the work reported in this paper.

## Data Availability

Data will be made available on request.

## Appendix A Supplementary data

Supplementary material Image 1
