# Supplementary material for: The authentication of Yanchi tan lamb based on lipidomic combined with particle swarm optimization-back propagation neural network
Source: Food Chem X. 2024 Nov 22;24:102031. doi: 10.1016/j.fochx.2024.102031 (PMC11629254; doi:10.1016/j.fochx.2024.102031)
Supplement: Supplementary file 1 — Supplementary material [file mmc1.docx]

**The authentication of Yanchi Tan lamb based on lipidomic combined with particle swarm optimization-back propagation neural network**

Qi Yang ^a^, Chongxin Liu ^a^, Muxuan Xu ^a^, Le Xu ^a^, Shaobo Li ^a^, Xiaochun Zheng ^a^, Dequan Zhang ^a^, Li Chen ^a, *^

*^a^* *Institute of Food Science and Technology, Chinese Academy of Agriculture Sciences, Key Laboratory of Agro-Products Quality and Safety Control in Storage and Transport Process, Ministry of Agriculture and Rural Affairs, Beijing 100193, China.*

*Corresponding authors*

***Li Chen^*^***

*E-mail: group2_chenli@126.com*

*Website: https://ifst.caas.cn/en/scientists/associateprofessors/262487.htm*

**Table S1** Sample information.

| **Variety** | **Origin** | **Sampling Company** |
| --- | --- | --- |
| Yanchi Tan lamb | Longitude 107°24′48″E, Latitude 37°47′21″N, Yanchi County, Ningxia Hui Autonomous Region, Ningxia, China | Ningxia Yanchi Tan Lamb Industry Development Group Co. Ltd |
| Jingyuan Tan lamb | Longitude 104°40′57″E, Latitude 36°34′38″N, Jingyuan County, Gansu Province, China | Gansu Senyang Agricultural and Animal Husbandry Science and Technology Development Limited Liability Company |
| Ertokqianqi Tan lamb | Longitude 107°29′4″E, Latitude 38°11′18″N, Ertokqianqi, Inner Mongolia Autonomous Region, China | Ningxia Yanchi Tan Lamb Industry Development Group Co. Ltd |

**Table S2** The identified volatile compounds and their odor descriptors in in Tan lamb from different regions.

| Compounds | Odor | NX（μg/kg） | | GS（μg/kg） | | IM（μg/kg） | |
| --- | --- | --- | --- | --- | --- | --- | --- |
|  |  | LT | KM | LT | KM | LT | KM |
| Hexanal | green | 55.13±2.33a | 58.28±1.53a | 44.95±1.15b | 40.27±3.52b | 10.47±0.65c | 12.36±1.16c |
| 4-Penten-1-ol | - | 0.69±0.04b | 0.87±0.09a | 0.72±0.07ab | 0.60±0.05b | - | - |
| (E)-2-Hexenal | green | 6.47±0.67bc | 7.6±0.53b | 27.83±2.04a | 29.47±2.81a | 2.68±0.37c | 2.22±0.27c |
| 2,3-Dimethyl-1-pentanol | green fruity | 6.94±0.55c | 7.10±0.93c | 10.91±1.41bc | 11.62±1.38b | 27.99±2.27a | 26.26±1.40a |
| Heptanal | oily | 9.72±0.89a | 9.12±0.73a | 5.30±0.56bc | 6.29±0.81b | 3.91±0.90c | 4.26±0.53bc |
| 1-Octen-3-ol | mushroom | 25.35±1.68c | 27.35±1.56bc | 53.80±2.67a | 31.10±1.03b | 3.57±0.64d | 2.90±0.39d |
| 3-Octanone | fruity | 43.57±3.61b | 42.03±2.86b | 54.67±1.96a | 48.93±2.41ab | 3.56±0.64c | 2.85±0.40c |
| 2-Ethyl-2-hexen-1-ol | green | 20.68±1.58b | 21.90±0.86b | 31.15±2.52a | 30.05±0.96a | 10.66±1.53c | 10.20±1.11c |
| Octanal | fatty | 6.56±0.52a | 6.28±0.48a | 2.83±0.33b | 2.63±0.58b | 1.02±0.14c | 0.86±0.14c |
| Formic acid octyl ester | green fruity | 1.74±0.54a | 1.15±0.17ab | 1.19±0.32ab | 1.02±0.14ab | 0.39±0.04b | 0.34±0.05b |
| (E)-2-Decenal | waxy orange | 4.89±0.22ab | 5.69±0.38a | 5.70±0.52a | 4.40±0.42b | 2.11±0.08c | 1.91±0.24c |
| 1-Nonanol | - | 0.8±0.02a | 0.8±0.04a | 0.89±0.01a | 0.84±0.04a | 0.39±0.03b | 0.46±0.03b |
| 2-Nonen-1-ol | - | 1.56±0.03d | 1.57±0.04d | 4.69±0.33c | 4.23±0.41c | 12.36±0.47b | 14.09±0.99a |
| 1-Decanol | - | 1.50±0.05a | 1.45±0.04a | 1.53±0.02a | 1.50±0.04a | 0.89±0.05b | 0.90±0.02ba |
| Nonanal | fatty | 27.63±1.89a | 27.62±1.26a | 11.28±0.63b | 11.75±0.36b | 0.83±0.03c | 0.79±0.02c |
| 2-Butyl-1-octanol | - | 0.46±0.03 | 0.44±0.06 | 0.40±0.03 | 0.47±0.01 | 0.42±0.03 | 0.39±0.03 |
| Decanal | - | 0.46±0.04c | 0.46±0.03c | 0.80±0.03a | 0.83±0.02a | 0.56±0.03b | 0.54±0.02bc |

Note: Each value is expressed as Mean ± Standard Error (S.E.); a-d Different letters indicate statistically significant differences based on a Duncan test at a level of significance of *p* < 0.05.

**Table S3** Abundance of different origins and parts of Tan lamb on each lipid class.

|  | **NX-LT** | **NX-KM** | **GS-LT** | **GS-KM** | **IM-LT** | **IM-KM** |
| --- | --- | --- | --- | --- | --- | --- |
| BA | 6.83×10^5^±4.32×10^4^ | 6.90×10^5^±3.19×10^4^ | 8.92×10^5^±7.87×10^4^ | 9.42×10^5^±5.22×10^4^ | 1.05×10^6^±2.47×10^5^ | 1.05×10^6^±2.19×10^5^ |
| CAR | 4.31×10^8^±2.62×10^7^b | 5.45×10^8^±3.79×10^7^a | 4.38×10^8^±2.33×10^7^b | 4.54×10^8^±2.65×10^7^b | 4.19×10^8^±2.39×10^7^b | 4.35×10^8^±1.19×10^7^b |
| Cer-AP | 1.16×10^6^±1.21×10^5^b | 9.78×10^5^±1.99×10^5^b | 2.14×10^6^±2.12×10^5^b | 4.35×10^6^±8.48×10^5^a | 1.53×10^6^±2.96×10^5^b | 1.76×10^6^±4.32×10^5^b |
| Cer-AS | 1.83×10^6^±6.49×10^4^ | 1.73×10^6^±9.66×10^4^ | 1.60×10^6^±5.93×10^4^ | 1.74×10^6^±6.10×10^4^ | 1.67×10^6^±6.55×10^4^ | 1.81×10^6^±8.54×10^4^ |
| Cer-NDS | 3.06×10^6^±1.18×10^5^a | 2.66×10^6^±1.23×10^5^b | 2.87×10^6^±1.02×10^5^ab | 2.82×10^6^±7.28×10^4^ab | 2.92×10^6^±6.58×10^4^ab | 2.65×10^6^±1.27×10^5^b |
| Cer-NP | 2.07×10^6^±1.60×10^5^ab | 1.56×10^6^±7.88×10^4^bc | 2.35×10^6^±2.49×10^5^a | 1.51×10^6^±1.38×10^5^c | 2.12×10^6^±1.75×10^5^a | 1.82×10^6^±1.94×10^5^abc |
| Cer-NS | 5.47×10^6^±3.74×10^5^ab | 6.06×10^6^±5.69×10^5^a | 6.03×10^6^±4.46×10^5^a | 5.96×10^6^±4.07×10^5^a | 4.29×10^6^±2.32×10^5^b | 4.86×10^6^±3.71×10^5^ab |
| CerP | 3.12×10^5^±3.20×10^4^a | 2.40×10^5^±1.65×10^4^abc | 2.36×10^5^±3.12×10^4^abc | 1.81×10^5^±1.94×10^4^c | 2.67×10^5^±1.95×10^4^ab | 2.26×10^5^±2.59×10^4^bc |
| Cho | 1.56×10^6^±4.37×10^4^b | 1.30×10^6^±3.56×10^4^c | 1.79×10^6^±4.70×10^4^a | 1.60×10^6^±7.31×10^4^b | 1.68×10^6^±3.88×10^4^ab | 1.59×10^6^±7.31×10^4^b |
| CoQ | 8.55×10^6^±6.10×10^5^ | 8.28×10^6^±5.97×10^5^ | 8.65×10^6^±1.17×10^6^ | 7.65×10^6^±1.29×10^6^ | 8.29×10^6^±5.02×10^5^ | 9.44×10^6^±7.16×10^5^ |
| DG | 2.15×10^6^±1.19×10^5^ab | 1.86×10^6^±1.43×10^5^b | 2.03×10^6^±1.21×10^5^ab | 2.29×10^6^±9.88×10^4^ab | 2.32×10^6^±1.65×10^5^a | 2.06×10^6^±1.65×10^5^ab |
| DG-O | 7.86×10^5^±9.59×10^4^ | 7.33×10^5^±7.67×10^4^ | 7.85×10^5^±8.98×10^4^ | 7.78×10^5^±8.11×10^4^ | 8.10×10^5^±9.39×10^4^ | 7.54×10^5^±8.97×10^4^ |
| Eicosanoid | 3.70×10^4^±3.36×10^3^ab | 2.68×10^4^±4.53×10^3^b | 6.33×10^4^±1.69×10^4^a | 2.96×10^4^±6.00×10^3^b | 6.12×10^4^±8.92×10^3^a | 3.07×10^4^±6.53×10^3^b |
| FFA | 2.91×10^7^±1.19×10^6^a | 2.69×10^7^±2.09×10^6^ab | 2.48×10^7^±7.80×10^5^b | 2.88×10^7^±8.07×10^5^a | 2.39×10^7^±8.21×10^5^b | 2.33×10^7^±7.16×10^5^b |
| HexCer-AP | 1.68×10^6^±7.63×10^4^b | 1.66×10^6^±1.07×10^5^b | 1.73×10^6^±6.79×10^4^b | 1.86×10^6^±1.19×10^5^ab | 2.02×10^6^±1.30×10^5^ab | 2.12×10^6^±1.41×10^5^a |
| HexCer-NS | 2.27×10^6^±3.64×10^5^bc | 2.13×10^6^±2.16×10^5^bc | 3.90×10^6^±3.61×10^5^a | 2.97×10^6^±3.86×10^5^b | 1.76×10^6^±1.16×10^5^c | 1.85×10^6^±2.23×10^5^c |
| LNAPE | 1.40×10^7^±1.49×10^6^ab | 1.55×10^7^±8.36×10^5^a | 1.10×10^7^±1.01×10^6^bc | 1.03×10^7^±1.41×10^6^c | 1.38×10^7^±7.91×10^5^ab | 1.30×10^7^±6.75×10^5^abc |
| LPA | 1.73×10^5^±6.73×10^3^ab | 1.84×10^5^±6.31×10^3^a | 1.60×10^5^±2.65×10^3^b | 1.75×10^5^±3.51×10^3^a | 1.60×10^5^±2.27×10^3^b | 1.61×10^5^±2.38×10^3^b |
| LPC | 2.56×10^7^±1.86×10^6^ab | 2.88×10^7^±2.12×10^6^ab | 2.34×10^7^±2.59×10^6^bc | 3.05×10^7^±1.65×10^6^a | 1.83×10^7^±1.69×10^6^c | 1.88×10^7^±1.40×10^6^c |
| LPC-O | 2.03×10^7^±2.19×10^6^b | 2.52×10^7^±2.55×10^6^ab | 1.96×10^7^±2.08×10^6^b | 3.22×10^7^±2.60×10^6^a | 1.74×10^7^±2.46×10^6^b | 1.86×10^7^±3.03×10^6^b |
| LPE | 4.02×10^6^±1.68×10^5^b | 4.56×10^6^±4.68×10^5^b | 4.69×10^6^±2.48×10^5^b | 6.16×10^6^±6.92×10^5^a | 4.35×10^6^±4.73×10^5^b | 4.28×10^6^±4.57×10^5^b |
| LPE-P | 1.82×10^6^±1.58×10^5^b | 2.90×10^6^±3.59×10^5^b | 2.01×10^6^±1.24×10^5^b | 4.28×10^6^±7.34×10^5^a | 1.94×10^6^±1.45×10^5^b | 2.26×10^6^±2.22×10^5^b |
| LPG | 1.10×10^6^±1.34×10^5^a | 9.87×10^5^±9.58×10^4^ab | 7.35×10^5^±9.47×10^4^b | 7.34×10^5^±8.96×10^4^b | 1.18×10^6^±1.11×10^5^a | 9.25×10^5^±1.01×10^5^ab |
| LPI | 6.10×10^5^±3.83×10^4^c | 9.12×10^5^±7.51×10^4^b | 9.11×10^5^±5.51×10^4^b | 1.52×10^6^±1.52×10^5^a | 7.66×10^5^±5.72×10^4^bc | 8.36×10^5^±6.87×10^4^bc |
| LPS | 1.31×10^5^±8.44×10^3^b | 2.12×10^5^±1.84×10^4^a | 1.60×10^5^±1.11×10^4^b | 2.35×10^5^±2.88×10^4^a | 1.40×10^5^±5.07×10^3^b | 1.56×10^5^±5.01×10^3^b |
| MG | 1.62×10^6^±1.90×10^4^ | 1.47×10^6^±1.11×10^5^ | 1.62×10^6^±8.91×10^4^ | 1.71×10^6^±3.40×10^4^ | 1.71×10^6^±4.13×10^4^ | 1.53×10^6^±1.04×10^5^ |
| MGDG | 5.91×10^5^±1.28×10^5^ab | 3.72×10^5^±7.24×10^4^b | 9.39×10^5^±1.36×10^5^a | 4.85×10^5^±2.10×10^5^b | 8.75×10^5^±2.87×10^4^a | 8.97×10^5^±8.31×10^4^a |
| PA | 2.66×10^7^±2.28×10^6^b | 3.16×10^7^±1.99×10^6^ab | 3.19×10^7^±2.05×10^6^ab | 3.92×10^7^±5.12×10^6^a | 3.10×10^7^±2.99×10^6^ab | 3.65×10^7^±3.42×10^6^ab |
| PC | 3.06×10^8^±1.94×10^7^abc | 3.67×10^8^±1.06×10^7^a | 2.62×10^8^±1.14×10^7^c | 2.94×10^8^±4.07×10^7^bc | 2.99×10^8^±2.03×10^7^abc | 3.49×10^8^±2.09×10^7^ab |
| PC-O | 7.09×10^8^±5.05×10^7^a | 6.69×10^8^±6.68×10^7^ab | 5.30×10^8^±9.01×10^7^ab | 5.12×10^8^±1.14×10^8^ab | 4.67×10^8^±5.85×10^7^ab | 4.52×10^8^±6.33×10^7^b |
| PE | 7.78×10^7^±6.63×10^6^ab | 8.21×10^7^±3.76×10^6^a | 6.48×10^7^±4.15×10^6^b | 6.49×10^7^±5.92×10^6^b | 7.05×10^7^±2.80×10^6^ab | 7.02×10^7^±2.96×10^6^ab |
| PE-O | 1.19×10^8^±6.74×10^6^ | 1.32×10^8^±4.89×10^6^ | 1.29×10^8^±9.16×10^6^ | 1.40×10^8^±1.49×10^7^ | 1.14×10^8^±4.26×10^6^ | 1.26×10^8^±4.90×10^6^ |
| PE-P | 2.24×10^8^±1.41×10^7^b | 2.68×10^8^±7.83×10^6^ab | 2.50×10^8^±1.31×10^7^ab | 2.90×10^8^±2.56×10^7^a | 2.56×10^8^±1.44×10^7^ab | 2.97×10^8^±1.81×10^7^a |
| PG | 3.95×10^7^±2.40×10^6^ab | 4.52×10^7^±1.13×10^6^a | 3.10×10^7^±2.24×10^6^d | 3.25×10^7^±3.49×10^6^cd | 3.76×10^7^±1.18×10^6^bc | 4.02×10^7^±1.59×10^6^ab |
| PI | 2.20×10^7^±2.11×10^6^b | 2.58×10^7^±8.02×10^5^ab | 2.24×10^7^±1.39×10^6^b | 2.30×10^7^±2.70×10^6^ab | 2.40×10^7^±1.74×10^5^ab | 2.76×10^7^±8.74×10^5^a |
| PMeOH | 4.31×10^5^±8.55×10^4^ | 4.37×10^5^±6.85×10^4^ | 3.68×10^5^±3.84×10^4^ | 3.88×10^5^±6.48×10^4^ | 3.22×10^5^±4.98×10^4^ | 3.29×10^5^±4.76×10^4^ |
| PS | 1.15×10^8^±8.80×10^6^ab | 1.25×10^8^±3.44×10^6^a | 9.89×10^7^±5.55×10^6^b | 9.68×10^7^±1.02×10^7^b | 1.22×10^8^±4.36×10^6^a | 1.25×10^8^±3.62×10^6^a |
| SHexCer | 7.51×10^5^±1.12×10^5^b | 7.37×10^5^±6.49×10^4^b | 1.10×10^6^±8.28×10^4^a | 1.03×10^6^±9.01×10^4^a | 4.93×10^5^±3.73×10^4^c | 5.73×10^5^±5.45×10^4^bc |
| SM | 6.47×10^7^±5.22×10^6^b | 6.42×10^7^±2.74×10^6^b | 7.28×10^7^±4.41×10^6^ab | 8.24×10^7^±6.55×10^6^a | 6.28×10^7^±2.79×10^6^b | 6.51×10^7^±3.45×10^6^b |
| SPH | 4.46×10^6^±1.68×10^5^a | 4.56×10^6^±3.63×10^5^a | 3.65×10^6^±2.95×10^5^b | 3.62×10^6^±2.15×10^5^b | 4.18×10^6^±1.52×10^5^bc | 4.27×10^6^±2.75×10^5^bc |
| TG | 1.15×10^9^±1.04×10^8^b | 7.92×10^8^±8.71×10^7^b | 1.19×10^9^±1.12×10^8^b | 2.03×10^9^±3.58×10^8^a | 1.29×10^9^±1.69×10^8^b | 1.34×10^9^±2.56×10^8^b |

Note: Each value is expressed as Mean ± Standard Error (S.E.); a-c Different letters indicate statistically significant differences based on a Duncan test at a level of significance of *p* < 0.05.

**Table S4** Model Selection Process for Identifying Yanchi and Non-Yanchi Tan Lambs.

| Number of  Variables | Important variable selected | Variance contribution rate | Cumulative variance contribution rate | Canonical correlation | Forecast Accuracy | Cross-validation Accuracy |
| --- | --- | --- | --- | --- | --- | --- |
| 11 | CAR1+CAR2+CAR3+CAR4+CAR5+CAR6+CAR7+CAR8+CAR9+CAR10+CAR11 | 100% | 100% | 0.945 | 100% | 94.4% |
| 10 | CAR1+CAR2+CAR3+CAR4+CAR5+CAR6+CAR7+CAR8+CAR9+CAR10 | 100% | 100% | 0.930 | 100% | 94.4% |
| 9 | CAR1+CAR2+CAR3+CAR4+CAR5+CAR6+CAR7+CAR8+CAR9 | 100% | 100% | 0.912 | 100% | 94.4% |
| 8 | CAR1+CAR2+CAR3+CAR4+CAR5+CAR6+CAR7+CAR9 | 100% | 100% | 0.894 | 97.2% | 91.7% |
| 7 | CAR1+CAR2+CAR3+CAR4+CAR5+CAR6+CAR7 | 100% | 100% | 0.889 | 97.2% | 91.7% |
| 6 | CAR1+CAR2+CAR3+CAR4+CAR5+CAR6 | 100% | 100% | 0.889 | 97.2% | 94.4% |
| 5 | CAR1+CAR2+CAR3+CAR5+CAR6 | 100% | 100% | 0.871 | 94.4% | 94.4% |
| **4** | **CAR1+CAR2+CAR3+CAR5** | **100%** | **100%** | **0.870** | **97.2%** | **94.4%** |
| 3 | CAR1+CAR3+CAR5 | 100% | 100% | 0.870 | 94.4% | 94.4% |
| 2 | CAR3+CAR5 | 100% | 100% | 0.826 | 88.9% | 88.9% |
| 1 | CAR5 | 100% | 100% | 0.826 | 88.9% | 88.9% |

**Table S5** Performance comparison of machine learning classification models.

| Models | BP | GA-BP | **PSO-BP** |
| --- | --- | --- | --- |
| Training set |  |  |  |
| Precision | 0.86 | 0.95 | **1.00** |
| Recall | 1.00 | 1.00 | **1.00** |
| Accuracy | 0.89 | 0.96 | **1.00** |
| Error rate | 0.11 | 0.04 | **0.00** |
| F1 | 0.92 | 0.97 | **1.00** |
| Test set |  |  |  |
| Precision | 0.63 | 0.80 | **1.00** |
| Recall | 1 | 0.80 | **1.00** |
| Accuracy | 0.63 | 0.75 | **1.00** |
| Error rate | 0.37 | 0.25 | **0.00** |
| F1 | 0.77 | 0.80 | **1.00** |


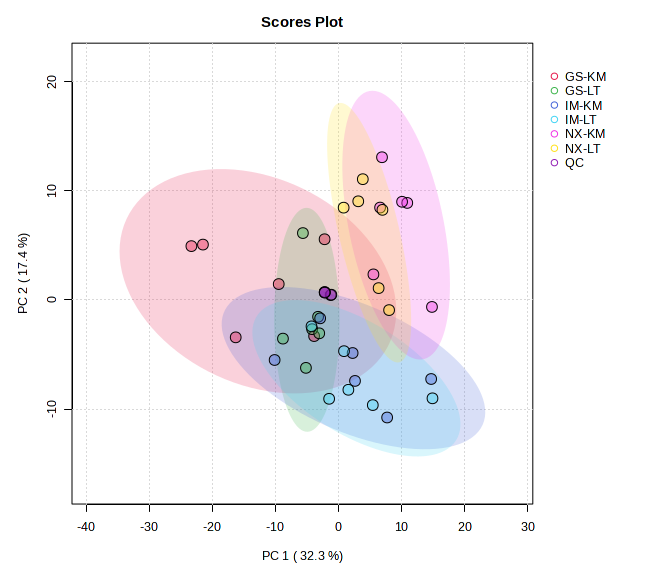


**Fig. S1.** Principal component analysis results for sample quality assessment.


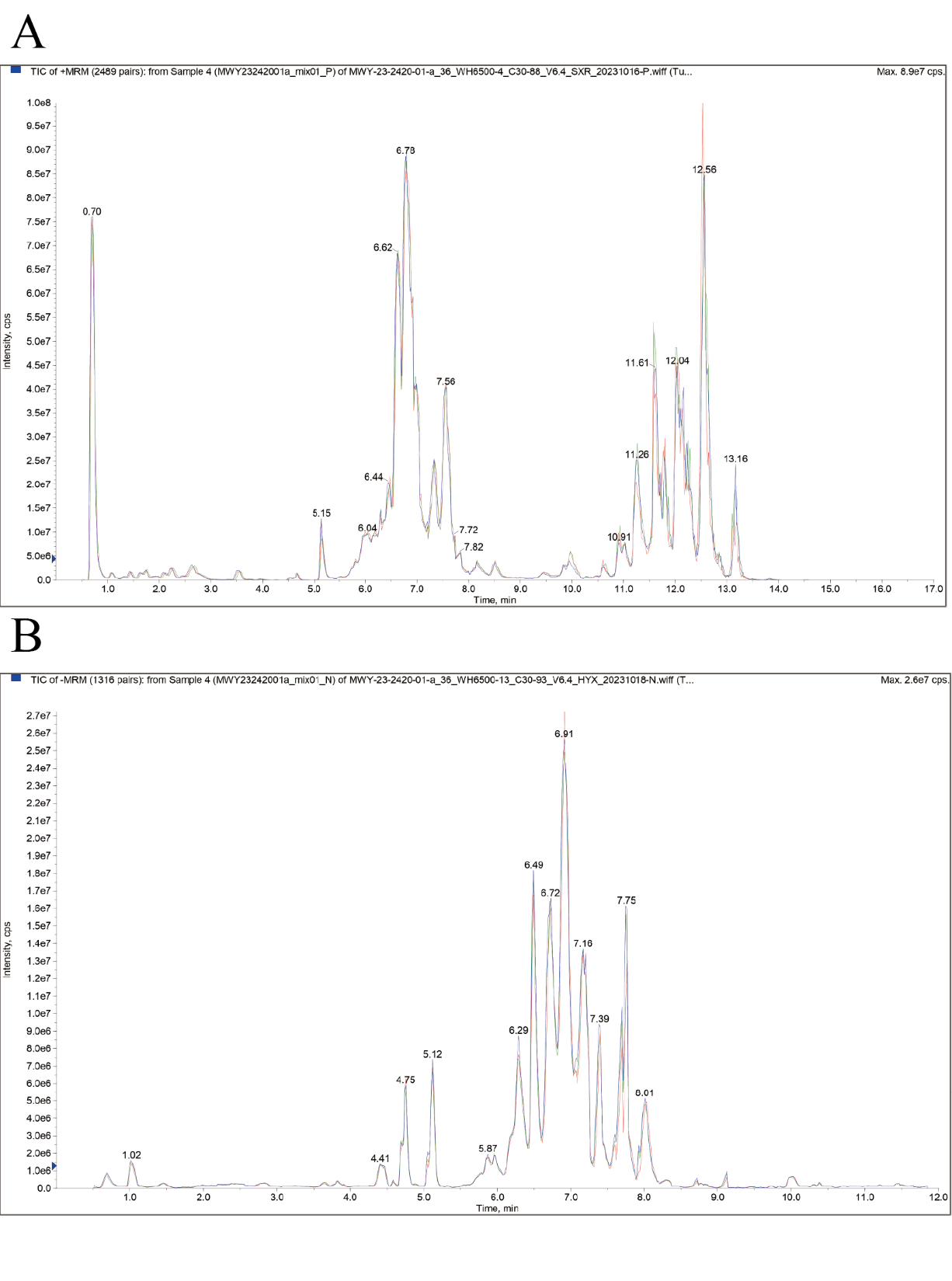
 **Fig. S2.** Total ion flow plots of the mixed sample in positive ion mode (A) and negative ion mode (B).
